# Mixed neuronal-glial tumor in the temporal lobe of an infant: a case report

**DOI:** 10.1186/1746-1596-8-164

**Published:** 2013-10-02

**Authors:** Hirohito Yano, Chiemi Saigoh, Noriyuki Nakayama, Yoshinobu Hirose, Masato Abe, Naoyuki Ohe, Michio Ozeki, Jun Shinoda, Toru Iwama

**Affiliations:** 1 Department of Neurosurgery, Gifu University Graduate School of Medicine, Gifu, Japan; 2 Department of Pathology, Gifu University Hospital, Gifu, Japan; 3 Department of Pathology, Osaka Medical College, Takatsuki, Japan; 4 Department of Diagnostic Pathology, School of Medicine Fujita Health University, Toyoake, Japan; 5 Department of Pediatrics, Gifu University Graduate School of Medicine, Gifu, Japan; 6 Chubu Medical Center for Prolonged Traumatic Brain Dysfunction, Department of Neurosurgery, Kizawa Memorial Hospital, Minokamo, Japan

**Keywords:** Neuronal tumor, Glioneuronal tumor, Mixed tumor, Temporal tumor, Infant, Epilepsy

## Abstract

**Background:**

Tumors that arise in the temporal lobes of infants and spread to the neural system are limited to several diagnoses. Herein, we present an infantile case of a temporal tumor showing neuronal and glial differentiation.

**Case presentation:**

The patient was a 9-month-old boy with low body weight due to intrauterine growth retardation. At 9 months after birth, he presented partial seizures. Computed tomography scanning revealed a mass (35 * 40 mm) in the left temporal lobe. Isointensity was noted on magnetic resonance T1-weighted images and fluid attenuation inversion recovery images. The tumor was heterogeneously enhanced with gadolinium. Positron emission tomography showed high methionine uptake in the tumor. During surgery, the tumor, which was elastic and soft and bled easily, was gross totally resected. A moderately clear boundary was noted between the tumor and normal brain parenchyma. Histologically, the tumor mainly comprised a ganglioglioma-like portion and short spindle cells at different densities. The former was immunohistochemically positive for some kinds of neuronal markers including synaptophysin. The spindle cells were positive for glial fibrillary acidic protein, but desmoplasia was not observed.

**Discussion:**

The tumor contained both neuronal and glial elements; the former were the main constituents of the tumor and included several ganglion-like cells. Because neuronal elements gradually transited to glial cells, a mixed neuronal-glial tumor was diagnosed.

**Virtual Slides:**

The virtual slide(s) for this article can be found here: http://www.diagnosticpathology.diagnomx.eu/vs/2045126100982604

## Background

Tumors that arise in the temporal lobes of infants and spread to the neural system include desmoplastic infantile ganglioglioma (DIG), pleomorphic xanthoastrocytoma (PXA), neuroblastoma (NB), and extraventricular neurocytoma (EVN). Herein, we present a case of a temporal tumor that spread to the neural system and discuss the tumor diagnosis in light of the clinical course, radiological findings, and histopathological findings.

## Case presentation

**Figure 1:**
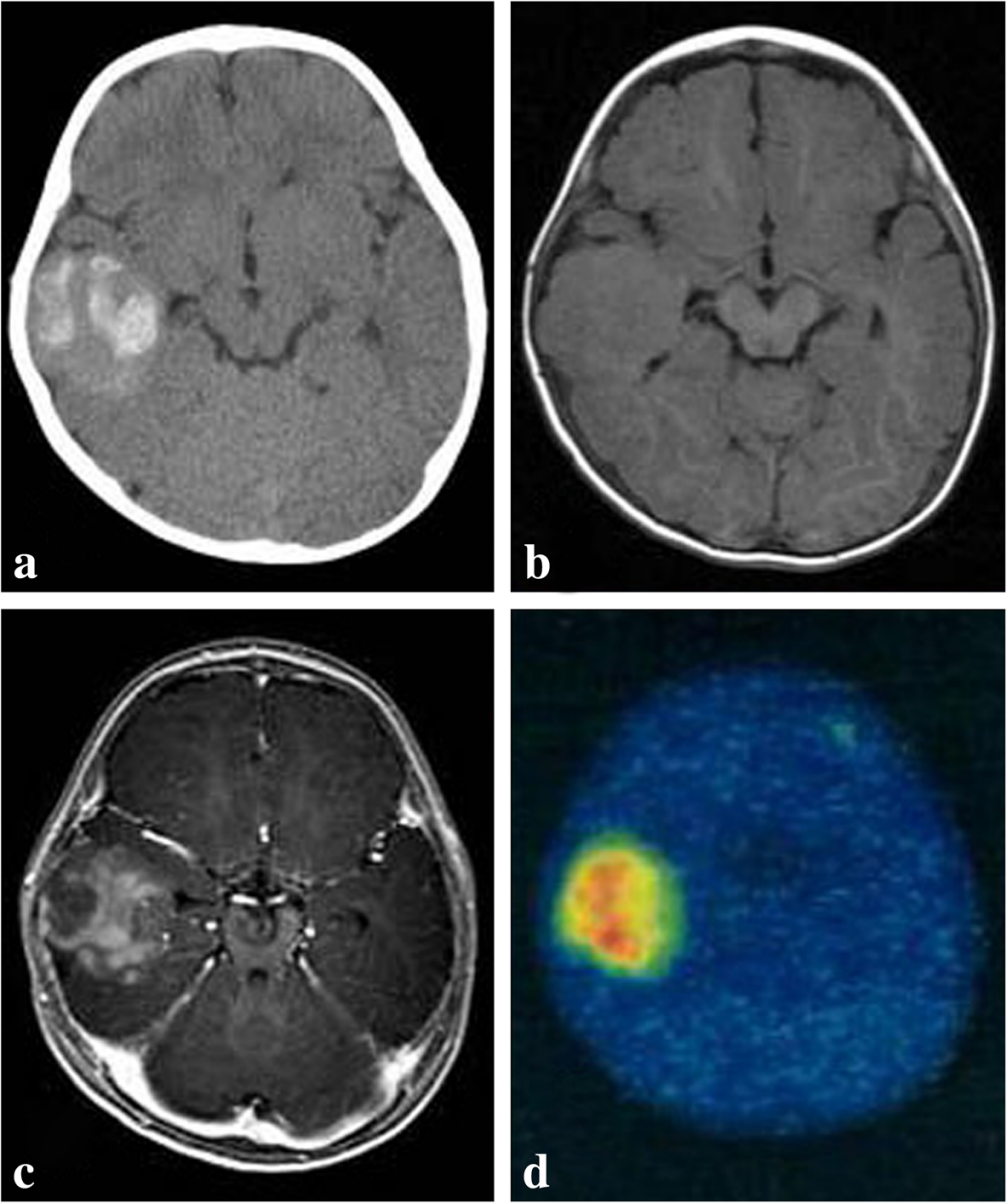
**Radiological imagings at admission. (a)** Computed tomography scan showing a calcified lesion in the right temporal lobe. Magnetic resonance imaging scans **(b)**: T1 plain-weighted image, **(c)**: Gadolinium-diethylenetriamine pentaacetic acid [Gd-DTPA]-enhanced images) showing a mass in the left temporal lobe. The mass was heterogeneously enhanced with Gd-DTPA. **(d)** Positron emission tomography showing high uptake of methionine in the lesion.

The patient was a 9-month-old boy who was born at 36 weeks 5 days of gestation with low body weight (2170 g) due to intrauterine growth retardation. At 9 months after birth, the patient was admitted to the hospital because of partial seizures. Upon admission, the patient’s level of consciousness was clear, and he had no neurological deficits. Computed tomography (CT) scanning revealed a mass lesion (35 × 40 mm) with calcification in the left temporal lobe (Figure 1a). Isointensity was noted on magnetic resonance (MR) T1-weighted images (WI), T2WI, and fluid attenuation inversion recovery images (Figure 1b). The tumor was heterogeneously enhanced with gadolinium (Gd) (Figure 1c). Cystic components were not observed. MR spectroscopy showed increased choline and lactate levels and decreased N-acetylaspartate levels in the region of interest. Positron emission tomography showed high methionine uptake in the tumor (Figure 1d). These findings suggested that the tumor had high cellularity with malignant alterations. Hence, a primitive neuroectodermal tumor was diagnosed. The patient underwent left temporal craniotomy, wherein the tumor was dissected and resected via the superior temporal sulcus. The tumor was gray in color and elastic-hard in texture; a moderately clear boundary was noted between the tumor and the normal brain parenchyma. The left inferior horn was opened by resection of the subependymal invasive tumor, which was elastic and soft and bled easily. The tumor was gross-totally resected. The patient’s postoperative course was generally good, but the convulsions persisted; hence, anticonvulsant therapy was continued. Two years after the surgery, the tumor showed no recurrence and the patient has been experiencing partial seizures monthly.

### Pathological examination

Formalin-fixed, paraffin-embedded tissue sections were examined via hematoxylin-eosin staining and immunohistochemistry. The primary antibodies and dilutions in buffer were as follows: mouse monoclonal anti-synaptophysin (Syn) antibody (1:50; Millipore), monoclonal anti-neurofilament protein (NFP) antibody (1:150; Dako), monoclonal anti-neuronal nuclear antigen (NeuN) antibody (1:100; Millipore), mouse monoclonal anti-tubulin, βIII isoform (TuJ1) antibody (1:200; Millipore), mouse monoclonal anti-glial fibrillary acidic protein (GFAP) antibody (1:500; Dako), rabbit polyclonal anti-olig 2 antibody (1:100; IBL), mouse monoclonal anti-isocitrate dehydrogenase (IDH)1 R132H antibody (1:20; Dianova), and monoclonal anti-Ki-67/MIB-1 antibody (1:50; Dako). For all antibodies, antigen retrieval was performed by autoclaving (121°C, 15 min). An Envision kit (Dako) provided secondary antibodies conjugated to dextran polymer and hydrogen peroxidase, and 3,3-diaminobenzidine was used as the chromogen. Silver impregnation was performed according to a previously described method of silver staining [1].

### Pathological findings

**Figure 2:**
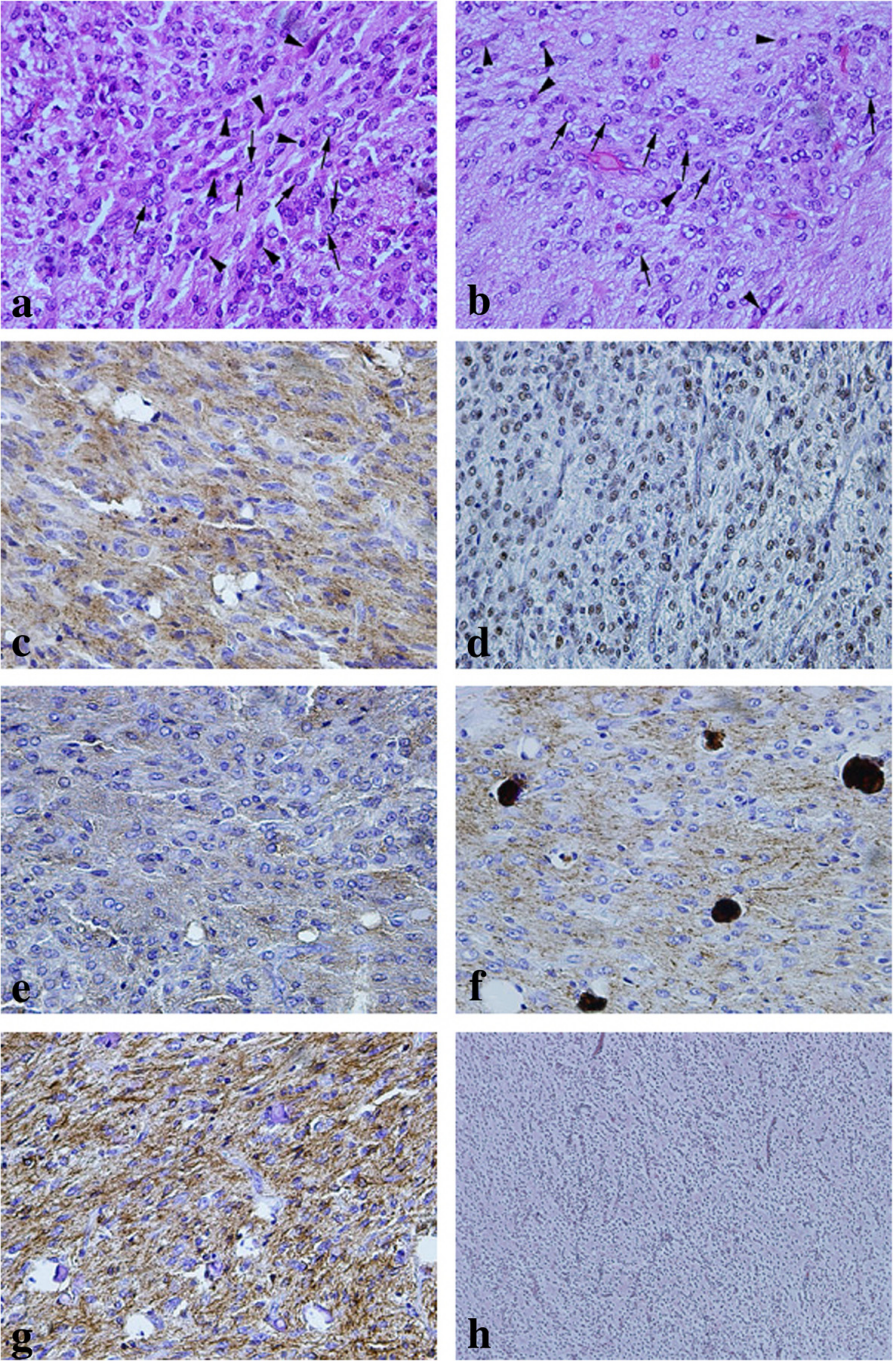
**Photomicrographs of hematoxylin-eosin staining and immunohistochemistry. (a,b)** Several cells with moderately large oval clear nuclei mimicking ganglioglioma (arrow) and eosinophilic spindle cells with small dark nucleus (arrowhead) were also observed. The large oval nuclei have marked nucleolar (arrow). These lesions were diffusely and strongly positive for **(c)** synaptophysin and **(d)** neuronal nuclear antigen. The cells stained positively for **(e)** tubulin, βIII isoform and **(f)** neurofilament in a spotty pattern. **(g)** On the other hand, spindle cells were positive for GFAP. **(h)** Silver impregnation staining revealed no desmoplasia. **(a**-**g**: ×400; **h**: ×100**)**.

**Figure 3:**
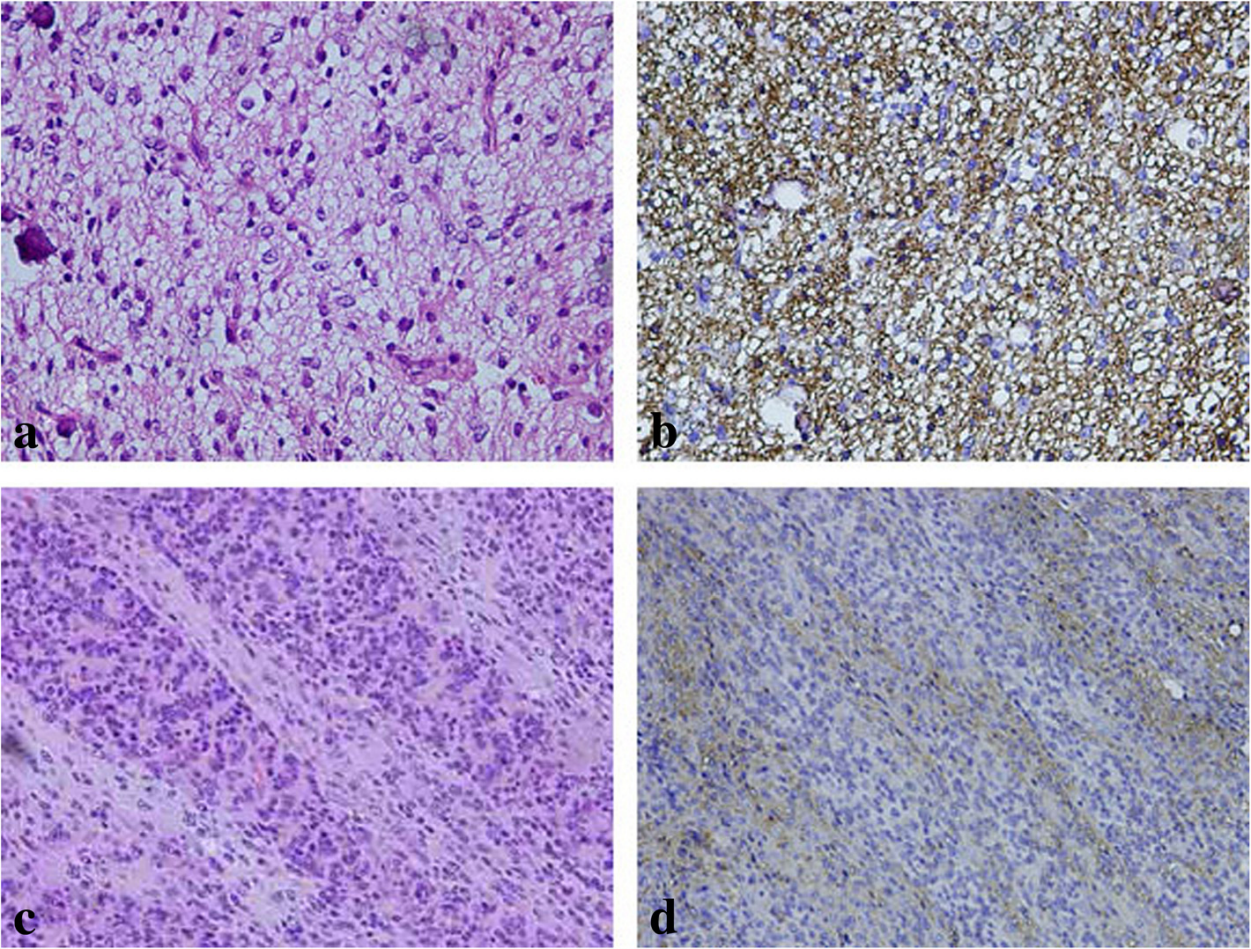
**Neurocytoma-like and neuroblastoma-like lesions. (a)** Hematoxylin-eosin (HE) staining showed an oligodendroglioma-like halo in the limited region. **(b)** The lesion corresponding to figure **(a)** was diffusely positive for glial fibrillary acidic protein. **(c)** HE staining showed tumor cells arranged in parallel rows. **(d)** The lesion corresponding to figure **(c)** was strongly positive for tubulin, βIII isoform in a parallel fashion. **(a**-**c**: ×400; **d**: ×200**)**.

The tumor mainly comprised a ganglioglioma-like portion and short spindle cells in different densities (Figure 2a, b). The ganglioglioma-like cells had large oval nuclei (Figure 2b). The ganglion-like cells were strongly immunohistochemically positive for Syn (Figure 2c), NeuN (Figure 2d), TuJ1 (Figure 2e) and NF (Figure 2f). These large cells partly proliferated in clusters (Figure 2b). Between these clusters of ganglion like cells, we found large numbers of spindle cells with moderately small nuclei, which were strongly positive for GFAP (Figure 2g). The tumor also contained a small oligodendroglioma-like lesion with a honeycomb appearance (Figure 3a). These lesions were positive for GFAP (Figure 3b) and olig 2 staining, but negative for IDH1 R132H. The MIB-1 labeling index was 5% in the area anchored by a large number of ganglioglioma-like cells; in contrast, the index was 2% in the area anchored by a large number of short spindle cells. Desmoplastic components were not observed by silver impregnation staining (Figure 2h). The tumor included an area that mimicked a primitive polar spongioblastoma pattern (Figure 3c), in which TuJ1 staining (Figure 3d) was strongly positive in a ladder-like fashion.

**Figure 4:**
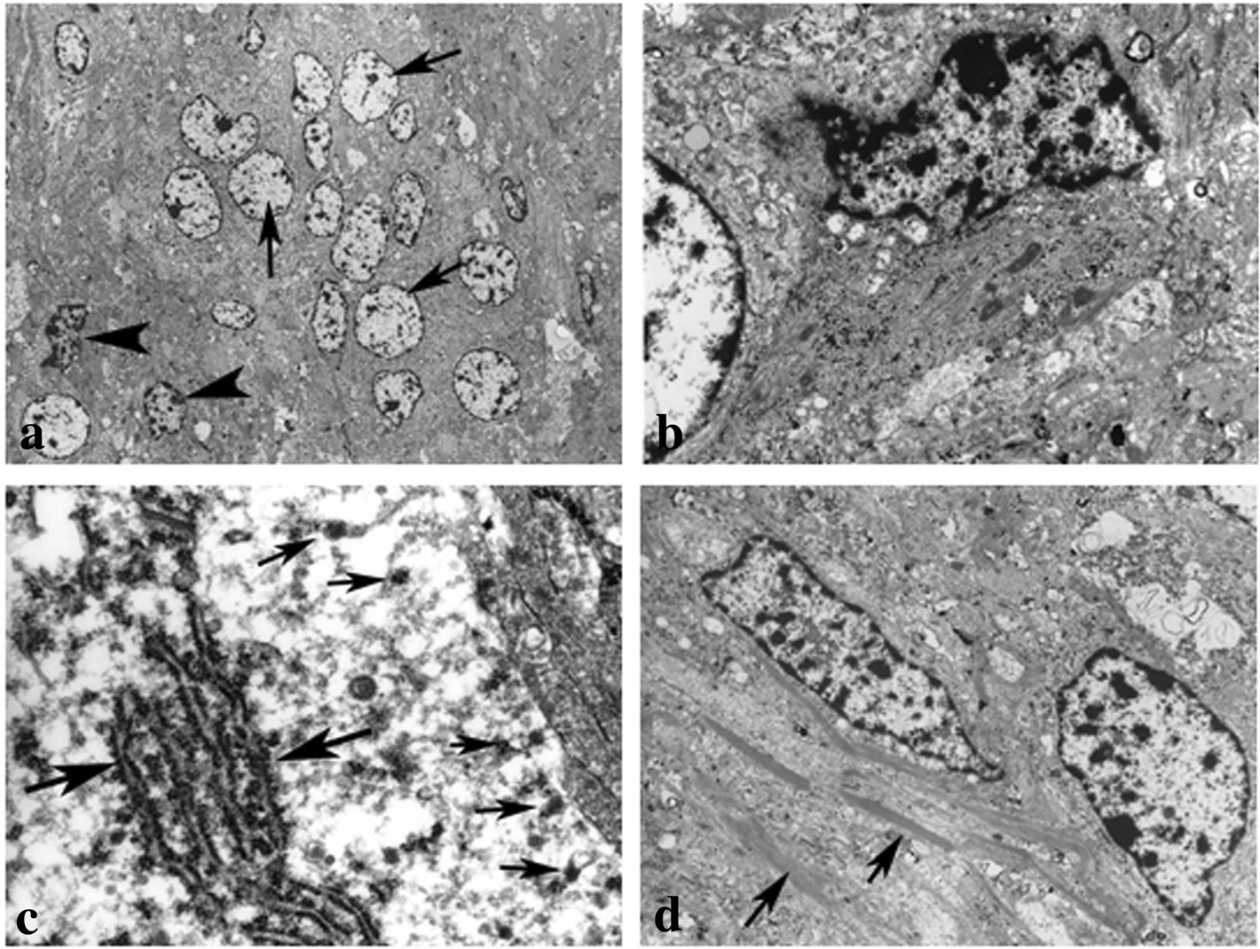
**Electron micrographs. (a)** Two types of tumor cells were observed: round or polygonal cells with round nuclei and moderately clear cytoplasm (arrow) and spindle cells with irregular nuclei and dark cytoplasm (arrowhead). **(b)** These cells were observed in contiguity with each other. **(c)** Abundant rough-surfaced endoplasmic reticulum (large arrow) and free ribosomes (small arrow) were observed in the cytoplasm of the former cells. **(d)** Some bundles of intermediate filaments (arrow) were noted in the cytoplasms of the latter cells. (**a**: ×800; **b**: ×4000; **c**: ×5.0000; **d**: ×5000).

Electron microscopic (EM) analysis revealed 2 types of tumor cells, round or polygonal cells with round nuclei and moderately clear cytoplasm and spindle cells with irregular nuclei and dark cytoplasm (Figure 4a, b). Abundant rough-surfaced endoplasmic reticulum and free ribosomes were observed as characteristic structures of the neuron (Figure 4c). A lot of intermediate filaments were easily observed in the spindle cells (Figure 4d). These findings suggested that the former type of tumor cells were neuron-like cells and the latter were astrocytic cells.

In summary, the tumor contained both neuronal and glial elements. The neuronal elements were the main constituents of this tumor and included several ganglion-like cells of different shapes. Because neuronal elements gradually transited to glial cells, a mixed neuronal-glial tumor was diagnosed.

## Discussion

The tumor in the present case was considered to have both neuronal and glial elements, according to the results of immunohistochemical and EM analyses. Especially, neuronal differentiation was observed primarily as presented in the immunohistochemistry for some kinds of neuronal markers. DIG, PXA, NB and EVN are possible diagnoses for a tumor with both neural and glial differentiation.

DIGs are rare primary neoplasms that account for 0.5–1.0% of all intracranial tumors [2]. DIG is a desmoplastic supratentorial neuroepithelial tumor that develops in patients whose ages range from 2 months to 59 years and occurs slightly more often in male individuals [3-6]. The large majority of these patients present with DIG within the first year of life [2]. Most patients with DIG present with new onset seizures [2,5,6], with or without focal neurological signs such as hemiparesis. Increased intracranial pressure and lethargy might be the only findings [7]. CT scans and MR images show large cystic tumors with enhancing solid components [6-9]. The frontal and temporal lobes are the most common sites of occurrence [2,6,7], wherein the tumor usually abuts the meningeal surface [10] and attaches to the dura. The tumors are firm and avascular with a dense desmoplastic component. There is no connection to the ventricular system. Microscopically, DIG shows evidence of glial and ganglionic differentiation, accompanied by an extreme desmoplastic reaction. In the present case, DIG was a possible differential diagnosis; however, no desmoplasia was histologically demonstrated. Radiological findings in this case did not show a cystic tumor, but rather a solid tumor that was exposed to the surface of the temporal lobe. Accordingly, we disregarded the diagnosis of DIG.

Secondary, PXA was also a possible diagnosis in the present case. These tumors occur most often in the temporal lobes of children or young adults [11-14], and associated seizures occur in up to 78% of cases [13]. PXA usually presents as a cyst with a superficially situated mural nodule. However, Yu et al. reported 8 solid-type cases out of 19 total PXA cases [15]. Microscopically, considerable pleomorphism, including spindle cells that transition through plump and/or polygonal cells to multinucleated giant cells, is observed. Endothelial proliferation and necrosis are absent. There have been reports of neuronal differentiation in PXA, according to immunohistochemical analysis [16-19]. Additionally, there have been several reports about the composition of PXA and ganglioglioma (PXA-GG) [20-22]. Sugita et al. summarized 17 cases of PXA-GG in which the patient ages ranged from 9 to 82 years. PXA-GG has not been reported in patients less than 1 year of age, as was the case for our patient. Thus, PXA could present with a divergent differentiation, as seen in the present case [19]. However, higher cellularity, dominant neuronal differentiation, and fewer pleomorphic findings of multinucleated giant cells noted in this case were different from the common type of PXA. For these reasons, we disregarded the diagnosis of PXA.

In recent years, there have some reports of molecular analyses associating with glioma development or progressions. It was reported that RTEL1 tagging single-nucleotide polymorphisms (SNPs) & haplotypes were identified to be associated with glioma development [23]. Furthermore, epidermal growth factor receptor and methylguanine-DNA methyltransferase (MGMT) promotor hypermethylation were reported to be associated with histological transformation and recurrence of gliomas. It seems like that the genetic alterations are early events in the development of glioma [24]. It is possible that these molecular markers may help the diagnosis of low grade glioma.

Cerebral NB, a rare embryonal tumor, was also considered a possible diagnosis. These tumors usually arise in the frontotemporal region of children and often occur early in the first decade of life. The incidence of NB in Mexican children was reported to be 3.8 per 1,000,000 children/year; the incidence of NB was the highest in children under 1 year of age, followed by those between 1 and 4 years of age (18.5 and 5.4 per 1,000,000 children/years, respectively) [25]. Histologically, homogenous and highly cellular arrangements with round to ovoid and hyperchromatic nuclei are observed. Varying numbers of Homer Wright rosettes are characteristic of these tumors. Rhythmic nuclear palisading, which produces parallel arrangements of cellular groups, might be observed. Neuronal differentiation can be detected by immunohistochemistry for neuronal markers such TuJ1, neurofilaments, and synaptophysin; however, glial differentiation is not demonstrated. In the present case, the rhythmic nuclear palisading pointed to this possible diagnosis; however, we disregarded it because of the presence of positive GFAP expression.

EVN was also a potential diagnosis. We previously reported a case of a 26-year-old woman who presented with atypical EVN, in which glial and neuronal differentiation was demonstrated by immunohistochemical analysis [26]. Histologically, EVN shows an isomorphous population of small cells with perinuclear halos that are arranged against a neuropil background. Ganglion-like cells might be intermixed with the neurocytic elements [27]. However, the patient in the present case had only a small number of oligodendroglioma-like halos in a limited portion. Because neurocytomas typically affect young adults, this was not applicable to the patient in our case with regard to the age of onset. Accordingly, we disregarded this diagnosis.

## Conclusion

We emphasized that the histological and EM study demonstrated both neuronal and glial differentiation in the present case. The observations of the present case did not completely correspond with any differential diagnosis with regard to epidemiological, radiological, and histological findings. This is a rare infantile case report of a tumor that consisted mainly of neuronal-glial tumor cells without desmoplasia.

## Consent

Written informed consent was obtained from the patient’s parents prior to the publication of this case report and accompanying images. A copy of the written consent is available for review by the Editor-in-Chief of this journal.

## Abbreviations

CT: Computed tomography; MR: Magnetic resonance; WI: Weighted images; Gd: Gadolinium; Syn: Synaptophysin; NFP: Neurofilament protein; NeuN: Neuronal nuclear antigen; TuJ1: Tubulin, βIII isoform; GFAP: Glial fibrillary acidic protein; IDH: Isocitrate dehydrogenase; EM: Electron microscopic; DIG: Desmoplastic infantile ganglioglioma; PXA: Pleomorphic xanthoastrocytoma; NB: Neuroblastoma; EVN: Extraventricular neurocytoma; GG: Ganglioglioma; SNP: Single-nucleotide polymorphism.

## Competing interests

The authors declare that they have no competing interests.

## Authors' contributions

HY was a major contributor in the writing of the manuscript. HY, SC, YH, NO, and MA were involved in pathological examination. NN and TI performed the surgery. MO conducted clinical examination. JS and TI were involved in the study design and coordination and helped to draft the manuscript. All authors have read and approved the final manuscript.
